# ANN: adversarial news net for robust fake news classification

**DOI:** 10.1038/s41598-024-56567-4

**Published:** 2024-04-03

**Authors:** Shiza Maham, Abdullah Tariq, Muhammad Usman Ghani Khan, Faten S. Alamri, Amjad Rehman, Tanzila Saba

**Affiliations:** 1 National Center of Artificial Intelligence, Al-Khawarizmi Institute of Computer Science, UET, Lahore, Pakistan; 2 Artificial Intelligence & Data Analytics Lab (AIDA) CCIS Prince Sultan University, 11586 Riyadh, Saudi Arabia; 3 Department of Mathematical Sciences, College of Science, Princess Nourah Bint Abdulrahman University, P.O. Box 84428, 11671 Riyadh, Saudi Arabia

**Keywords:** Fake news, Adversarial training, Figurative language, Transformers, FGSM, BERT, Longformer, Information technology, Computer science

## Abstract

With easy access to social media platforms, spreading fake news has become a growing concern today. Classifying fake news is essential, as it can help prevent its negative impact on individuals and society. In this regard, an end-to-end framework for fake news detection is developed by utilizing the power of adversarial training to make the model more robust and resilient. The framework is named "ANN: Adversarial News Net," emoticons have been extracted from the datasets to understand their meanings concerning fake news. This information is then fed into the model, which helps to improve its performance in classifying fake news. The performance of the ANN framework is evaluated using four publicly available datasets, and it is found to outperform baseline methods and previous studies after adversarial training. Experiments show that Adversarial Training improved the performance by 2.1% over the Random Forest baseline and 2.4% over the BERT baseline method in terms of accuracy. The proposed framework can be used to detect fake news in real-time, thereby mitigating its harmful effects on society.

## Introduction

The spread of fake news has become a significant concern in recent years due to its potential to manipulate public opinion, create social unrest, and undermine democratic processes. Fake news refers to deliberately fabricated or misleading information presented as factual. It can be disseminated through various mediums, including social media, news websites, and even traditional media outlets. The rapid and prevalent spread of false news has the potential to cause significant harm to society, including the ability to impact the outcome of political events such as elections.

In the modern digital age, where there are countless venues for exchanging data and the potential for fake news or misinformation to proliferate, the broad issue of fake news is exceedingly challenging to address. Artificial Intelligence development has made it a more significant problem by enabling the creation and dissemination of fake news by artificial bots^1^. According to a study conducted by researchers at New York University in 2018, false news stories are 70% more likely to be shared on Twitter than true news stories. The study analyzed the spread of over 4.5 million tweets and found that false news stories were more likely to elicit an emotional response, which made them more likely to be shared^2^. The problem is critical since most individuals trust what they read online, and those who are inexperienced or new to digital media might be easily tricked. Deception is another issue that might occur because of spam or malignant messages. Therefore, it is convincing enough to recognize the problem, reduce burglary, political turmoil, and suffering, and oppose the propagation of false information.

There have been many research efforts in the field of fake news classification. These efforts range from manual fact-checking to developing advanced Machine Learning (ML) algorithms. In Ref.^3^, the authors provided a comprehensive review of the existing techniques for fake news detection, including ML algorithms, social network analysis, and Deep Learning (DL) based approaches. It also highlighted the challenges and opportunities in this field. Reference^4^ presented a hybrid approach that combines Convolutional Neural Networks (CNNs) and Recurrent Neural Networks (RNNs) to detect fake news on Twitter. The authors achieved high accuracy in detecting fake news by combining word embeddings, character embeddings, and other text features. Moreover, a hierarchical attention network for detecting fake news is proposed in a research study^5^. The method utilized attention mechanisms to learn which parts of the news article are most important in making a classification decision. The authors achieved considerable accuracy in detecting fake news using article-level and sentence-level attention mechanisms. Recent studies have shown that fake news classification models developed in the past may be less accurate now, as social media users have become more sophisticated in their use of language. Slang words, special characters, and emotes are increasingly used in comments and posts, making it more difficult for these models to detect fake news accurately.

To address this issue, our methodology takes these factors into account. It is done by extracting the emotes from the datasets and then making a dictionary to pass to the models. Ultimately, developing more effective fake news detection methods is critical for ensuring that social media platforms remain a trusted source of information for users worldwide. By leveraging the latest advancements in AI and ML, we can help prevent the spread of false and misleading information and promote greater transparency and accountability in online communication. The primary focus of this paper is to classify fake news accurately. Four datasets are utilized to achieve this, and the performance of five ML and six DL algorithms, such as BERT, RoBERTa, DeBERTa, and Longformer, are evaluated.

Perturbed samples are generated for training the model on clean and perturbed data using the FGSM method. Notably, this dynamic approach added noise that improved model performance rather than compromising it. Additionally, the emotes dictionary is extracted to enhance VADER’s capabilities. In this study, the main research contributions are given below:

- Evaluate the performance of five ML and six DL algorithms, including BERT, RoBERTa, DeBERTa, and Longformer, for fake news classification.
- The extraction of the emotes dictionary is performed to enhance VADER’s capabilities for better lexicon annotation.
- The use of the FGSM method generates perturbed samples and uses them to enhance model performance.
- This study enhances fake news detection by incorporating slang words and emoticons into the model, while employing adversarial training for improved generalizability, outperforming baseline methods.

The paper’s remaining sections are structured as follows: Section "Literature survey" offers a literature survey focusing on the classification of fake news. Section "Methodology" outlines the methodology employed in the study, followed by Sect. "Results and discussion", which details the experimental settings, results, and analysis, and Sect. "Limitations" concludes the research.

## Literature survey

Fake news has been an emerging problem in the era of social broadcasting and online news outlets. The proliferation of fake news can lead to severe consequences, such as misinformation, manipulation, and social unrest. Researchers have been developing various techniques for detecting and classifying fake news to address this issue. Current machine learning (ML)-based approaches for detecting fake news rely on various news features. However, detecting fake news is challenging due to missing or unavailable information at the initial stage of news dissemination. This literature survey aims to review the contemporary advances in classifying fake news.

Consequently, it is tough to identify fake news accurately in its initial stages. To overcome this constraint, a new methodology is presented in Ref.^6^ for detecting misleading digital media posts early by categorizing news dissemination networks. Each news story's propagation path is represented as a multivariate time series, where each tuple is a numeric vector representing user traits. The model included recurrent and convolutional networks that captured global and local variations of user attributes, respectively. The model accurately identified fake news in only 5 min after the news circulated. Study^7^ used a computational framework incorporating geometric and probabilistic ML algorithms to combat fake stories. Also, the effectiveness of two distinct vectorizers, count and TF-IDF, are evaluated by comparing their results to determine which vectorizer performed best for identifying fake news. Several models are employed to identify the misleading content, including Naive Bayes, Support Vector Machines (SVM), Logistic Regression, and decision tree classification methods. Based on the simulation's findings, SVM using the TF-IDF provided the most accurate forecast. The research work proposed in Ref.^8^ employed an ML ensemble method to classify news articles automatically. This research investigated various textual characteristics that differentiate real news from fake news. These attributes are used to train multiple ML methods, which are then combined using diverse ensemble techniques. The experimental analysis demonstrated that the suggested approach outperformed solitary learners. Moreover, it is necessary to implement a system for fact-checking rumors and claims, especially those that gain widespread attention before being deflated by reliable sources. Fake news has been identified and categorized using a variety of ML methods. These methods are constrained in their accuracy, though. Reference^9^ used a Random Forest (RF) classifier to ascertain whether a news article is fake. To accomplish this, 23 textual characteristics are selected from the dataset, and four feature selection methods are utilized to pick the best 14 features out of 23. The intended model is compared with other benchmark methods using the best features. The experimental results demonstrated that the suggested framework surpasses state-of-the-art ML algorithms, such as GBM and Ada Boos, regarding categorization accuracy. Reference^10^ developed classifiers to categorize a specific news item as real or fraudulent using various ML methods. Various individual ML techniques like K-nearest neighbors (KNN) and Decision trees, as well as pre-built ensembled models like random forest and gradient boosting and custom ensemble classifiers like Stacking and Maximum Voting Classifier, are used for this. The study achieved a promising level of accuracy in classifying news as real or fake by combining three ML classifiers, specifically KNN, SVM, and Logistic Regression, into a distinct-ensembled model.

Moreover, a vital task in fake news classification is to adopt a bidirectional training scheme, which can effectively model the relevant information in fake news while enhancing the classification accuracy by capturing the semantic and long-range sentence dependencies. To address this^11^, presented a novel approach called FakeBERT, which employed a Deep Learning (DL) model based on BERT along with parallel blocks of a single-layer deep Convolutional Neural Network (CNN) having distinct kernel sizes and filters. This combination effectively addressed the problem of vagueness, which is a significant challenge in natural language interpretation. Reference^12^ introduced a novel approach for discerning authentic and counterfeit news articles using a hybrid BiLSTM and Self-Attention model. The hybrid BiLSTM model and self-attention and other pertinent layers effectively identified and classified fraudulent news articles from the original ones. The suggested model outperformed current DNN, BiGRU, LSTM, and Logistic regression models regarding validation accuracy, as evidenced by the comparative analysis. Reference^13^ developed a methodology that utilized a combination of convolutional neural networks and long short-term recurrent neural network models to identify and categorize misleading information disseminated through Twitter posts. The proposed system employed a DL technique and accomplished a significant accuracy rate. Furthermore, this approach had the ability to recognize essential characteristics connected to fabricated news articles without any prior comprehension of the area of expertise. Reference^14^ aimed to identify fake news on Romanian online sites using ML algorithms. Furthermore, the investigation included comparing outcomes obtained from different DL techniques, such as RNN that utilized LSTM and gated recurrent unit cells, CNN, and a pre-trained Romanian BERT model called RoBERTa. To assess the effectiveness of these methods, classical classification algorithms, namely Naïve Bayes and SVM, are also considered. CNN emerged as the most effective technique, surpassing the performance of traditional classification and BERT methods.

Reference^15^ explored the use of DL techniques and transformer approaches to identify the trustworthiness of Arabic news articles. Unlike previous studies that relied on manually crafted features, this study leveraged the power of DL and transformer models to extract and analyze the underlying patterns and information in the news data. The impact of news length on model performance is also examined by testing the models on different datasets that vary in average length. The findings revealed that the transformer-based model outperformed the DL model across all three datasets. Notably, the description dataset proved to be the most effective at accurately classifying news articles, likely due to the similarity in average length between real and fake news. On the other hand, the current suggested solutions for identifying fake news on centralized platforms primarily focus on analyzing the news content and not considering the location of the news. Reference^16^ proposed a region-based distributed approach for detecting fake news, which operates in a mobile crowdsensing environment. The framework chose workers based on their accessibility in a particular area, who oversee gathering and sharing the news with the closest edge node. The pre-processing and identification of fake news are carried out locally at the edge node. A pre-trained BERT model is employed for the identification process, which attains a considerable accuracy rate. Reference^17^ introduced a hybrid RNN and SVM model to identify real and fake news. The textual data, which included news articles and comments, was first encoded into numerical feature vectors using a bidirectional gated recurrent unit RNN. These encoded features were then passed to an SVM with a radial basis function kernel for classification. Tests on a real-world dataset provided promising outcomes and showed that the suggested methodology performed better than cutting-edge techniques.

A novel technique for regularizing the fine-tuning of Transformer-based encoders for text classification problems is provided in Ref.^18^. The model’s word embedding matrix is perturbed to provide adversarial examples, and contrastive learning is used to train the model to learn noise-invariant representation using clean and adversarial examples. They run experiments on various GLUE benchmark tasks and three intent classification datasets to gauge the effectiveness of their strategy. Their research showed that Roberta Large also performed 1–2% better than the Roberta Large baseline. In ref.^19^, the authors proposed a novel method called Consistent Regularization for Adaptation Learning (CRAL) for performing domain adaptation. The approach involved creating two distinct shared latent spaces, conducting domain alignment for each space, and penalizing any inconsistencies between the two alignments regarding predictions for unlabeled data. To ensure consistent regularization for the CRAL method, virtual adversarial training (VAT) is used with entropy minimization. The experimental results demonstrated that the CRAL method outperformed existing state-of-the-art techniques on two different Multi-Domain Text Classification (MDTC) benchmarks. Table 1 presents a critical review, analysis & comparison of the state of the art.

**Table 1:** Fake news classification: a comparison of benchmark datasets.

Reference	Year	Approaches	Purpose	Dataset	Evaluation measures	Evaluation values	Limitations
^6^	2018	CNN, RNN	Detecting misleading digital media posts	News stories are retrieved from Weibo, Twitter15, and Twitter16 social media sites	Accuracy	85% and 92% accuracy were achieved on Twitter and Sina Weibo in only 5 min after the news started circulating	The effectiveness of the proposed model that integrates semi-supervised learning and PU-learning techniques needs to be tested and validated, as it may have limitations in dealing with the constantly evolving landscape of social media and fake news
^7^	2019	Geometric and probabilistic ML algorithms (Naïve Bayes, SVM, Decision tree, logistic regression), neural network	Comparison of various ML algorithms for identifying fake news	The dataset was taken from Kaggle, which contains news articles collected from RSS feeds	Accuracy	SVM using the TF-IDF provided the most accurate forecast with a 92.8% accuracy	This study only considered news content for classification, which may limit the accuracy of the results. Additional factors such as the news title, source, and engagement statistics could improve classification accuracy. Therefore, the use of multiple attributes should be explored to enhance classification accuracy
^8^	2020	ML methods (Logistic regression, SVM, multilayer perceptron, KNN), ensemble learners (random forest, bagging ensemble classifier, boosting ensemble, Voting ensemble classifier)	Classify news articles by identifying patterns in textual data	The dataset was a collection of news articles from multiple domains retrieved from the World Wide Web	Accuracy, precision, recall, and F1-score	Compared to the individual learners, the ensemble learners have demonstrated superior performance across all performance metrics	Key sources that are involved in the spread of fake news need to be identified
^9^	2022	Random forest classifier	To ascertain whether a news article is fake	ISOT dataset	Accuracy	The suggested framework surpasses state-of-the-art ML algorithms by an accuracy of 96.42%	This study is only restricted to ML methods
^10^	2023	Individual ML methods (KNN, decision tree), In-built ensembled methods (random forest, gradient boosting), and custom ensemble classifiers (stacking, maximum voting algorithms)	To categorize a specific news item as real or fraudulent	Datasets of fake and true news are utilized	Accuracy	Combining three separate ML models, namely KNN, SVM, and Logistic Regression, into a custom ensembled model, this paper has achieved a classification accuracy of 91.5% in distinguishing between true and fake news	The scope of this study is limited exclusively to ML methods
^11^	2021	BERT-based deep convolutional approach	To detect fake news in social media	Real-world fake news dataset	Accuracy, cross entropy loss, false positive rate, and false negative rate	The suggested model achieved an accuracy of 98.90%	It did not utilize a hybrid approach incorporating content, context, and temporal-level information from news articles for binary and multi-class real-world fake news datasets
^12^	2022	Hybrid BiLSTM and self-attention model	Discern authentic and counterfeit news articles	Fake News dataset	Precision, recall, F1-score	Suggested model Outperformed current DNN, CNN, BiGRU, CNN + BiLSTM, LSTM, and Logistic regression models with an accuracy of 98.65%	The imbalanced dataset is the suggested model's primary drawback
^13^	2021	Hybrid CNN-RNN model	Identify and categorize misleading information disseminated through Twitter posts	ISO and FA-KES News dataset	Accuracy, precision, recall, and F1-score	The proposed model showed promising results using both datasets	In this study, only CNN and RNN architectures are considered More neural network structures are needed to be explored
^14^	2022	ML algorithms (Naïve Bayes, SVM), DL techniques (RNN with LSTM and GRU cells, CNN), and RoBERT	Identify fake news on Romanian online news sites	Romanian News dataset	Accuracy, precision, recall, and F1-score	CNN showed the best performance with an accuracy of 98.20%, surpassing traditional classification and BERT methods	This work is only limited to Romanian news
^15^	2023	LSTM, GRU, BiLSTM, BiGRU, CNN, DeepCNN, AraBERT, AraELECTRA, AraGPT2, QaRiB, ARBERT, MARBERT and zero-shot models	Identify the trustworthiness of Arabic news articles	ArabicFakeNews dataset	Accuracy, precision, recall, and F1-score	The transformer-based model outperformed the DL model. AraBERTv2 achieved the highest accuracy, precision, and f1-score of 97% on the description dataset among all other transformer architectures	It is only restricted to Arabic news content
^16^	2022	Pre-trained BERT model	Detect fake news in a region-based distributed approach	FakeNewsNet dataset	Precision, recall, and F1-score	The model achieved an accuracy of 91%	The distributed framework further needs to be optimized in the mobile crowdsensing environment
^17^	2021	A hybrid model combining RNN with Bidirectional GRU and SVM	Identify real and fake news	FakeNewsNet dataset	Accuracy, precision, recall, and F1-score	Suggested methodology performed better than cutting-edge techniques	The limitation of Support Vector Machines (SVM) is that their performance depends on the feature vector's size. In this case, the minimum size of the feature vector was restricted to 512 units, which is the output of the GRUs
^18^	2022	Perturbation of word embedding matrix and contrastive learning using transformers such as BERT and RoBERTa	GLUE benchmark tasks and three intent classification datasets	Accuracy	The method demonstrates an improvement of 1.7% on average over BERTLarge and 1.3% over RoBERTaLarge. On intent classification tasks, the fine-tuned RoBERTaLarge outperforms the RoBERTaLarge baseline by 1% on the entire test sets and 2% on the more challenging test sets	Regularizing Transformer-based encoders for text classification problems	Modest perturbations to input vector entries may not be appropriate for sparse high-dimensional inputs
^19^	2022	CRAL (consistent regularization for adaptation learning) and VAT (virtual adversarial training) with entropy minimization	Two MDTC (multi-domain text classification) benchmarks	Accuracy	88% and 90% on both datasets	Adversarial training for specific domain adaptation	Accuracy is compromised in an unseen domain

## Methodology

**Figure 1:**
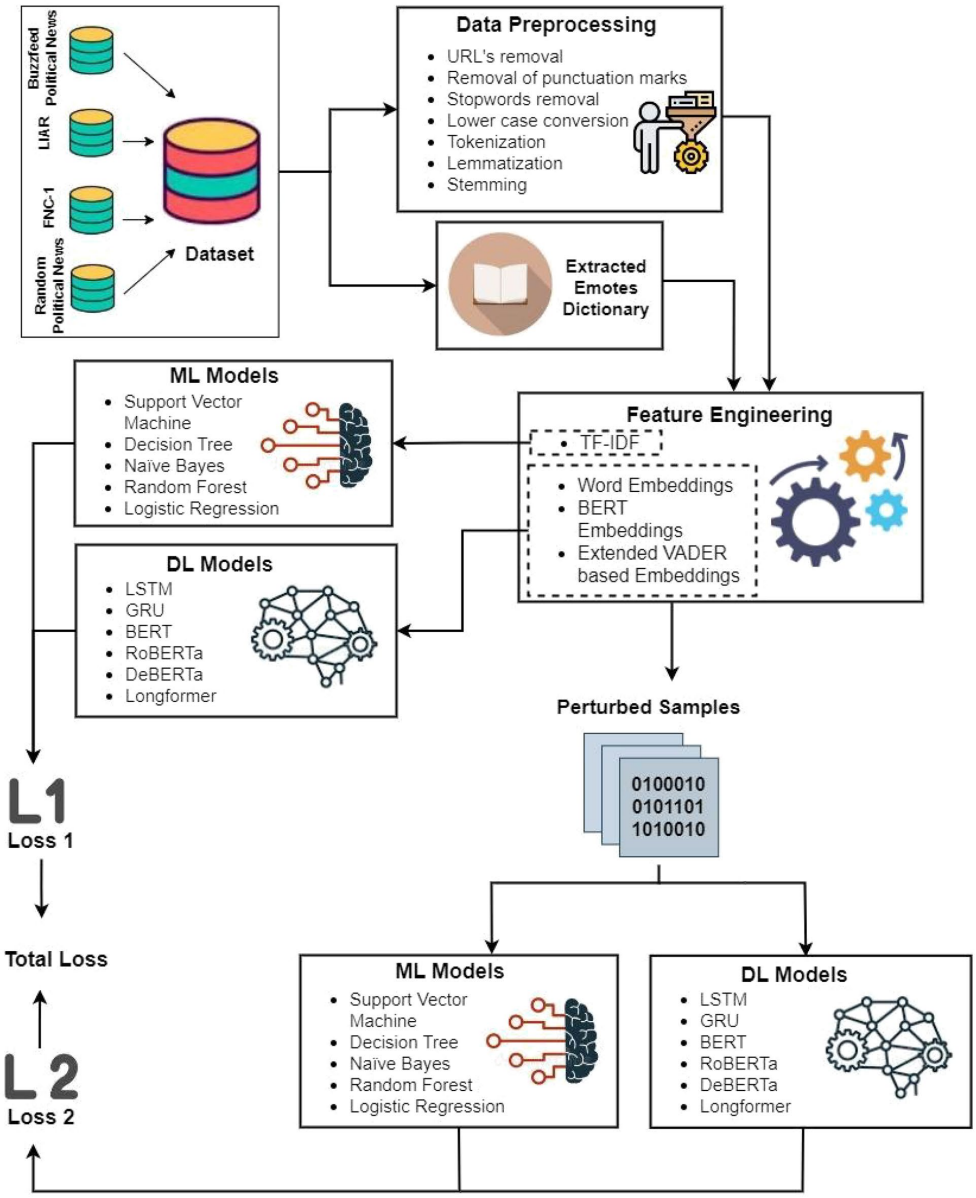
Flow diagram of ann: adversarial news net.

**Figure 2:**
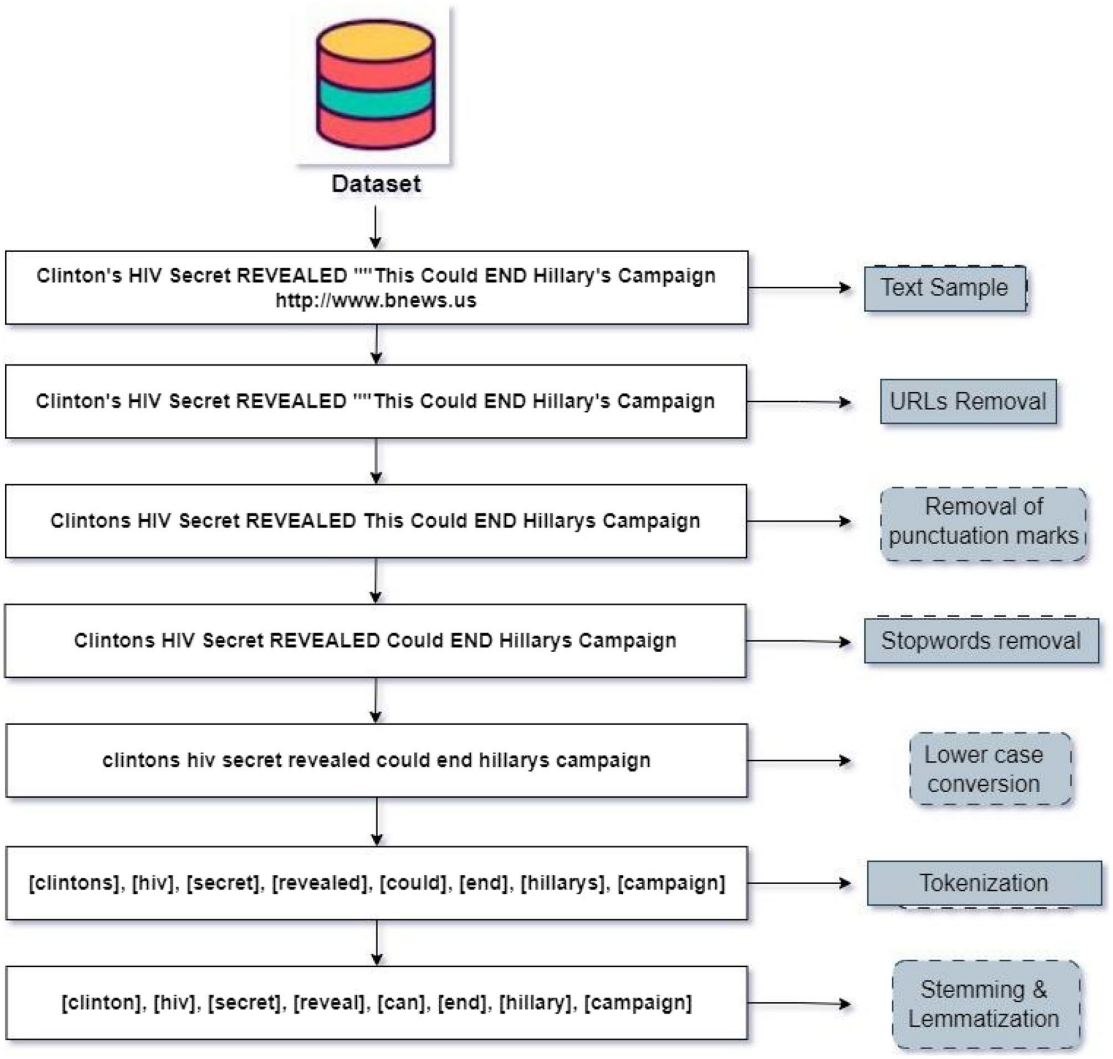
Preprocessing steps performed on the datasets.

As shown in Fig. 1 for fake news classification, the proposed methodology encompassed a sequence of intricate steps, commenced with gathering four publicly available datasets. To prepare the dataset for analysis, many preprocessing techniques are employed as elucidated in Fig. 2.

Additionally, a dictionary of emotes and slang words is extracted from the data, and polarity is ascribed to each emote based on its significance. After this, steps of feature engineering, training of models before adversarial training, and training of algorithms after adversarial training are carried out. This research study utilized six DL algorithms involving BERT, RoBERTa, DeBERTa, Longformer, GRU, LSTM, and five ML algorithms.

### Preprocessing

The first step in the proposed methodology is preprocessing. This step involved cleaning the data and removing irrelevant information, such as URLs, user names, and hashtags. The text is tokenized into words and stop words are removed from the text to reduce noise in the dataset.

### Extraction of emotes and slang words from the dataset

The second step is the extraction of emotes and slang words from the dataset. Emotes often convey emotions and can be essential for detecting fake news. Similarly, slang words, such as "lol" and "omg," can also be used to indicate the tone of the message. Therefore, these features are extracted from the dataset to improve the model's accuracy.

### Feature engineering

In the subsequent step, feature engineering is done by applying TF-IDF vectorization for the machine learning (ML) models while extracting word embeddings, BERT embedding, and extended Vader-based embeddings for the deep learning (DL) models. Word Embedding techniques used for feature engineering and their justification is given below in the section “Word Embedding Techniques Utilization and Justification**”.**

### Training of models before adversarial training

The fourth step in the proposed methodology is the training of models before adversarial training. The models are trained using BERT, RoBERTa, DeBERTa, Longformer, GRU, and LSTM. These models are then used as a baseline for comparison with the models trained after adversarial training.

### Training of algorithms after adversarial training

The fifth step in the proposed methodology is the training of algorithms after adversarial training. In this step, adversarial training is utilized to enhance the model's performance. Adversarial samples are generated by perturbing the original data and retraining the model on the perturbed data. These models are then used to classify fake news and compare their performance with the models trained before adversarial training. The models are also trained by using five machine learning algorithms such as Random Forest, Decision Tree, Support Vector Machine, Naive Bayes, and Logistic Regression. To obtain statistical representations of the input text, two transformer models, BERT and Longformer, are utilized as transformer models. These models are fine-tuned on publicly available datasets, with or without noise, for fake news classification.

### Adversarial training

Adversarial training generates adversarial samples by perturbing the inputs to misclassify them by the model. The Fast gradient sign method (FGSM) used to generate perturbed examples. Once adversarial examples are generated, the model is trained on a pair of perturbed and clean examples. The amount of perturbation generated using the FGSM method are represented as,

1 $$p=-\varepsilon sign \left(\nabla {X}_{i}L \left({f}_{\theta }\left({x}_{i}+r\right), {y}_{i}\right)\right),$$

where $L \left({f}_{\theta }\left({x}_{i}+r\right), {y}_{i}\right)$ represents the cross-entropy loss function, ${f}_{\theta }$ represents the neural network parameterized by θ, and ε is the hyperparameter to control the amount of perturbation. By merging these ideas, the overall score of the classification model is improved. In order to create adversarial examples, perturbation is introduced to the embedding matrix for each input text instead of directly modifying the input itself. This approach lets the model simultaneously on clean and adversarial examples.

## Results and discussion

### Dataset description

In this study, four publicly available datasets are utilized, namely the Random political news dataset^20^, the Buzzfeed political news dataset^20^, the LIAR dataset^21^, and the FNC-1 dataset^22^. The datasets consisted of articles, news reports, and online posts related to politics. The Random political news dataset consisted of 300 news articles, with 150 real and 150 fake news articles. The Buzzfeed political news dataset comprised 202 news articles, of which 106 are real news articles, and 96 are fake news articles. The LIAR dataset consisted of 2248 fake articles and 1894 real articles. The FNC-1 dataset included 3677 articles labeled as agree and 839 articles labeled as disagree. The stats of the dataset are given in Table 2.

**Table 2:** Dataset summary.

Dataset	Domain	Category	Number of news
Random political news	Politics	Real news	150
		Fake news	150
Buzzfeed political news	Politics	Real news	106
		Fake news	96
LIAR	Politics	False	2248
		True	1894
FNC-1	Politics	Agree	3677
		Disagree	839

### Word embedding techniques utilization and justification

This study employed a comprehensive suite of word embedding techniques^23^ namely TF-IDF, Word2Vec, BERT Embeddings, and Extended VADER, tailored to the unique nuances of political datasets for robust analysis and classification of fake news content.

#### TF-IDF

First of all this study utilized the power of TF-IDF (Term Frequency-Inverse Document Frequency) stands as a fundamental technique in natural language processing (NLP), particularly for its simplicity in word representation and computational efficiency. TF-IDF analysis revealed terms such as "election fraud," "misinformation," or "propaganda" exhibit heightened importance and frequency in fake news articles compared to legitimate sources.

#### WORD2VEC

By incorporating Word2Vec embeddings, the classification models gained the ability to capture varied semantic relationships and contextual nuances prevalent in news. Word2Vec embeddings encoded semantic associations between terms such as "political candidate" and "scandal," or "policy" and "controversy," thereby facilitating the identification of misleading or sensationalized narratives. This contextual understanding enhanced the models' capability to discern subtle linguistic cues indicative of deceptive practices or biased articles within the dataset.

#### BERT embeddings

The integration of BERT-based embeddings augmented the classification models with contextualized word representations, enabling a deeper understanding of the complex linguistic structures inherent in news content. BERT embeddings captured the contextual variations surrounding terms such as "government policies," "public opinion," or "partisan rhetoric," thereby facilitating more accurate classification of fake news articles based on their semantic coherence and syntactic structures.

#### Extended VADER

Most prominent and successive word embedding used in this study is Extended VADER. In the realm of news, where emotive language and varied sentiments play a significant role, Extended VADER emerges as a vital tool for sentiment analysis and word embedding. By augmenting the VADER lexicon with specific emoticons and slang terminologies, such as "lol," "omg," or "wtf," this study enhanced the sentiment analysis capabilities of the classification models. Extended VADER accurately discerned the sentiment orientations of deceptive or inflammatory content, thereby facilitating more complex and accurate classification of fake news articles based on their emotive content and rhetorical strategies.

### Experimental setup

The experimental setup for the fake news classification involved five distinct steps, all of which are conducted using the Google Colab Pro Plus version. As outlined in the dataset chapter, four datasets are utilized with an 80:20 training-to-testing ratio. A batch size of 32 is used, alongside a learning rate of 0.0001 and a decay rate of 0.00001. The DL and transformers algorithms are trained for 70 epochs with early stopping. Additionally, the model is trained twice, once with perturbed samples and once without perturbed samples. The overall hyperparameter details for all ML, DL and transformer-based models are provided in Tables 3, 4 and 5 respectively.

**Table 3:** Dataset summary.

Model	Parameters
SVM	C = 1.0, Kernel = rbf, degree = 3, gamma = scale, random_state = 42
Linear SVM	C = 1.0, Kernel = Linear, verbose = 0, random_state = 42
Decision tree	criterion = 'gini', min_samples_split = 2, min_samples_leaf = 1, ccp_alpha = 0.0
Naive Bayes	priors = None, var_smoothing = 1e-09
Logistic regression	penalty = 'l2', C = 1.0, random_state = 48, max_iter = 100, verbose = 0, l1_ratio = None
Random forest	random_state = 48, verbose = 0, ccp_alpha = 0.0
K-nearest neighbor	n_neighbors = 5, weights = 'uniform', leaf_size = 30, p = 2, metric = 'minkowski', metric_params = None

**Table 4:** Dataset summary.

Model	Parameters
LSTM	loss = binary_crossentropy, optimizer = adam, dropout = 0.2, recurrent_dropout = 0.5, activation = sigmoid, epochs = 50, batch_size = 32, learning_rate = 0.0001
GRU	loss = binary_crossentropy, optimizer = adam, dropout = 0.2, activation = sigmoid, epochs = 50, batch_size = 32, learning_rate = 0.0001

**Table 5:** Dataset summary.

Model	Parameters
BERT	max_len = 250, epochs = 50, batch_size = 32, dropout = 0.2/0.5, learning_rate = 0.0001, hidden_size = 12, num-attention_heads = 12, model = bert-base-cased
Longformer	attention_window = 1024, num_layers = 12, hidden_size = 768, dropout = 0.2/0.5, max_position_embeddings = 4096, num_attention_heads = 12
DeBERTa	max_len = 250, epochs = 50, batch_size = 32, dropout = 0.2/0.5, learning_rate = 0.0001, hidden_size = 12, num-attention_heads = 12, model = deberta-base
RoBERTa	max_len = 250, epochs = 50, batch_size = 32, dropout = 0.2/0.5, learning_rate = 0.0001, hidden_size = 768, num_attention_heads = 12, model = roberta-base

### Evaluation measures

#### Precision

Precision refers to the ratio of true positive instances, i.e. the instances that are correctly predicted as positive, to all instances predicted as positive. This metric measures the accuracy of positive predictions made by the model.

2 $$Precision=\frac{True \,\,Positive}{True\,\, Positive+False \,\,Positive},$$

#### Recall

Recall measures the ratio of true positive instances, i.e. correctly predicted positive instances, to all true positive instances present in the dataset. This metric evaluates the model's ability to identify positive instances from the dataset accurately.

3 $$Recall=\frac{True \,\,Positive}{True\,\, Positive+False \,\,Negative},$$

#### F1-score

The F1-score is a metric that considers the model's precision and recall to evaluate its overall accuracy. It is calculated as the harmonic mean of precision and recall, providing a more balanced assessment of the model's performance than accuracy alone.

4 $$F1-Score=2*\frac{\left(Precision * Recall\right)}{Precision + Recall},$$

#### Accuracy

Accuracy refers to the proportion of correctly classified instances out of all the instances in the dataset. It is a measure of how well the model performs overall.

5 $$Accuracy=\frac{True\,\, Positive+True\,\, Negatives}{Total\,\, Instances}.$$

The Eqs. (2), (3), (4) and (5) utilize various terms to quantify model performance. The term "True Positives" denotes the number of correctly predicted positive instances, while "False Positives" signifies the number of instances predicted as positive but were, in fact, negative. "False Negatives" represents the number of instances predicted as negative but were actually positive, and "True Negatives" denotes the number of correctly predicted negative instances. Finally, the term "Total Instances" signifies the overall number of instances present in the dataset.

### Results

#### Baseline results

Four distinct datasets are utilized and produced results that outperformed the state-of-the-art. Specifically, on the "Random Political News" dataset, SVM demonstrated precision of 81.3%, recall of 82.2%, F1 score of 82%, and accuracy of 83.3%. Similarly, the Decision Tree yielded a precision of 80.1%, recall of 79%, F1 score of 81.8%, and accuracy of 81.7%. Additionally, Logistic Regression obtained a precision of 83%, recall of 82%, F1 score of 81.7%, and accuracy of 82.88%. Naive Bayes achieved a precision of 84.2%, recall of 83.9%, F1 score of 84.1%, and accuracy of 84%, while Random Forest attained a precision of 89%, recall of 91.3%, F1 score of 90%, and accuracy of 90.7%.

**Figure 3:**
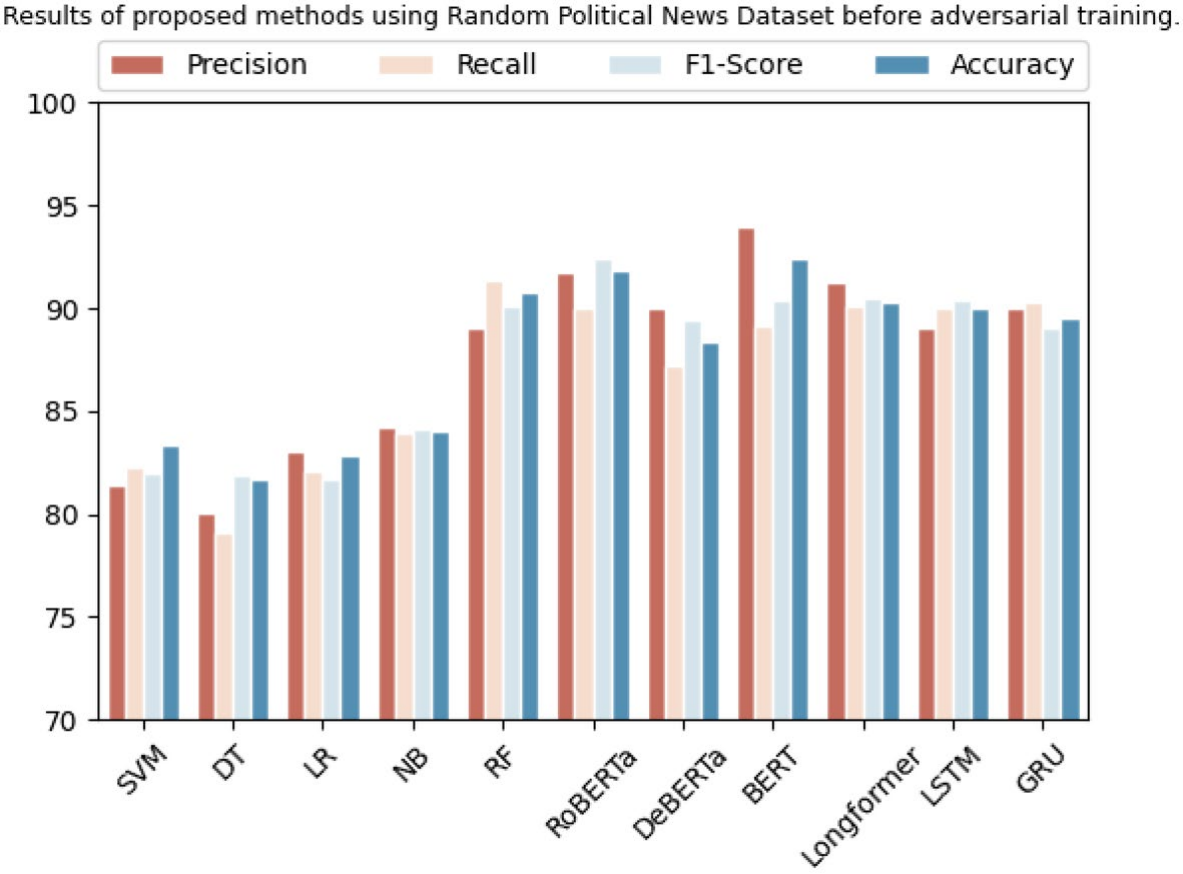
Graphical representation of results on baseline methods using random political news dataset.

In contrast, Transformer-based models like RoBERTa demonstrated strong performance with a precision of 91.7%, recall of 90%, F1 score of 92.4%, and accuracy of 91.8%. Similarly, DeBERTa achieved a precision of 90.0%, recall of 87.2%, F1 score of 89.4%, and accuracy of 88.33%. BERT exhibited high precision with a score of 93.9%, although its recall was slightly lower at 89.1%. Its overall F1 score was 90.3%, and its accuracy was 92.43%. Furthermore, Longformer achieved a precision of 91.2%, recall of 90.1%, F1 score of 90.4%, and accuracy of 90.3%. Finally, LSTM and GRU models yielded precision scores of 89% and 90%, recall scores of 90% and 90.3%, F1 scores of 90.3% and 89%, and accuracy scores of 90% and 89.5%, respectively. The overall results are presented in Table 6 and Fig. 3 shows their graphical representation.

**Table 6:** Results on proposed methods using random political news dataset before adversarial training.

Dataset	Methodology	Precision	Recall	F1-score	Accuracy
Random political news	SVM	81.3%	82.2%	82.0%	83.3%
	Decision tree	80.1%	79.0%	81.8%	81.7%
	Logistic regression	83.0%	82.0%	81.7%	82.88%
	Naive Bayes	84.2%	83.9%	84.1%	84.0%
	Random forest	89.0%	91.3%	90.0%	90.7%
	RoBERTa	91.7%	90.0%	92.4%	91.8%
	DeBERTa	90.0%	87.2%	89.4%	88.33%
	BERT	93.9%	89.1%	90.3%	92.43%
	Longformer	91.2%	90.1%	90.4%	90.3%
	LSTM	89.0%	90.0%	90.3%	90.0%
	GRU	90.0%	90.3%	89.0%	89.5%

**Figure 4:**
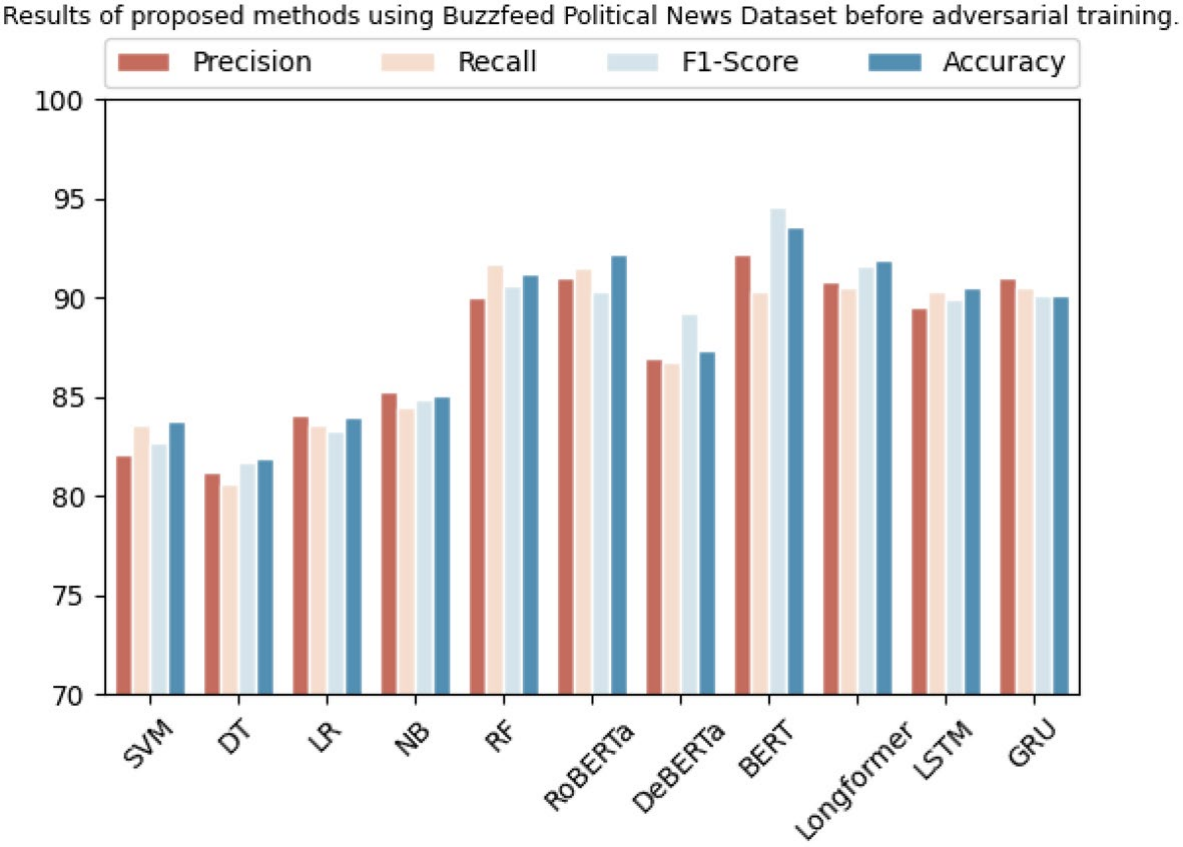
Graphic representation of results on baseline methods using Buzzfeed political news dataset.

The BuzzFeed political news dataset yielded favorable results across various algorithms, which can be seen in Table 7. SVM demonstrated a precision rate of 82.0%, a recall rate of 83.5%, an F1-score of 82.7%, and an accuracy rate of 83.7%. Decision Tree yielded a precision rate of 81.2%, a recall rate of 80.5%, an F1-score of 81.6%, and an accuracy rate of 81.9%. Logistic Regression yielded a precision rate of 84.0%, recall rate of 83.5%, F1-score of 83.3%, and accuracy rate of 83.9%. Naive Bayes achieved a precision rate of 85.2%, a recall rate of 84.5%, an F1-score of 84.9%, and an accuracy rate of 85.1%. Random Forest yielded a precision rate of 90.0%, recall rate of 91.7%, F1-score of 90.6%, and accuracy rate of 91.1%. Additionally, the Transformer-based algorithms demonstrated exceptional results. RoBERTa yielded a precision rate of 91.0%, a recall rate of 91.5%, an F1-score of 90.3%, and an accuracy rate of 92.2%. DeBERTa demonstrated a precision rate of 87.0%, a recall rate of 86.8%, an F1-score of 89.2%, and an accuracy rate of 87.3%. BERT achieved a precision rate of 92.2%, a recall rate of 90.3%, an F1-score of 94.5%, and an accuracy rate of 93.6%. Longformer yielded a precision rate of 90.8%, recall rate of 90.5%, F1-score of 91.6%, and accuracy rate of 91.9%. Lastly, LSTM and GRU demonstrated a precision rate of 89.5% and 91.0%, recall rate of 90.3% and 90.5%, F1-score of 89.9% and 90.8%, and accuracy rate of 90.5% and 90.1%, respectively. The graphical representation of these results is shown in Fig. 4.

**Table 7:** Results of proposed methods using Buzzfeed political news dataset before adversarial training.

Dataset	Methodology	Precision	Recall	F1-score	Accuracy
Buzzfeed political news	SVM	82.0%	83.5%	82.7%	83.7%
	Decision tree	81.2%	80.5%	81.6%	81.9%
	Logistic regression	84.0%	83.5%	83.3%	83.9%
	Naive Bayes	85.2%	84.5%	84.9%	85.1%
	Random forest	90.0%	91.7%	90.6%	91.1%
	RoBERTa	91.0%	91.5%	90.3%	92.2%
	DeBERTa	87.0%	86.8%	89.2%	87.3%
	BERT	92.2%	90.3%	94.5%	93.6%
	Longformer	90.8%	90.5%	91.6%	91.9%
	LSTM	89.5%	90.3%	89.9%	90.5%
	GRU	91.0%	90.5%	90.8%	90.1%

**Figure 5:**
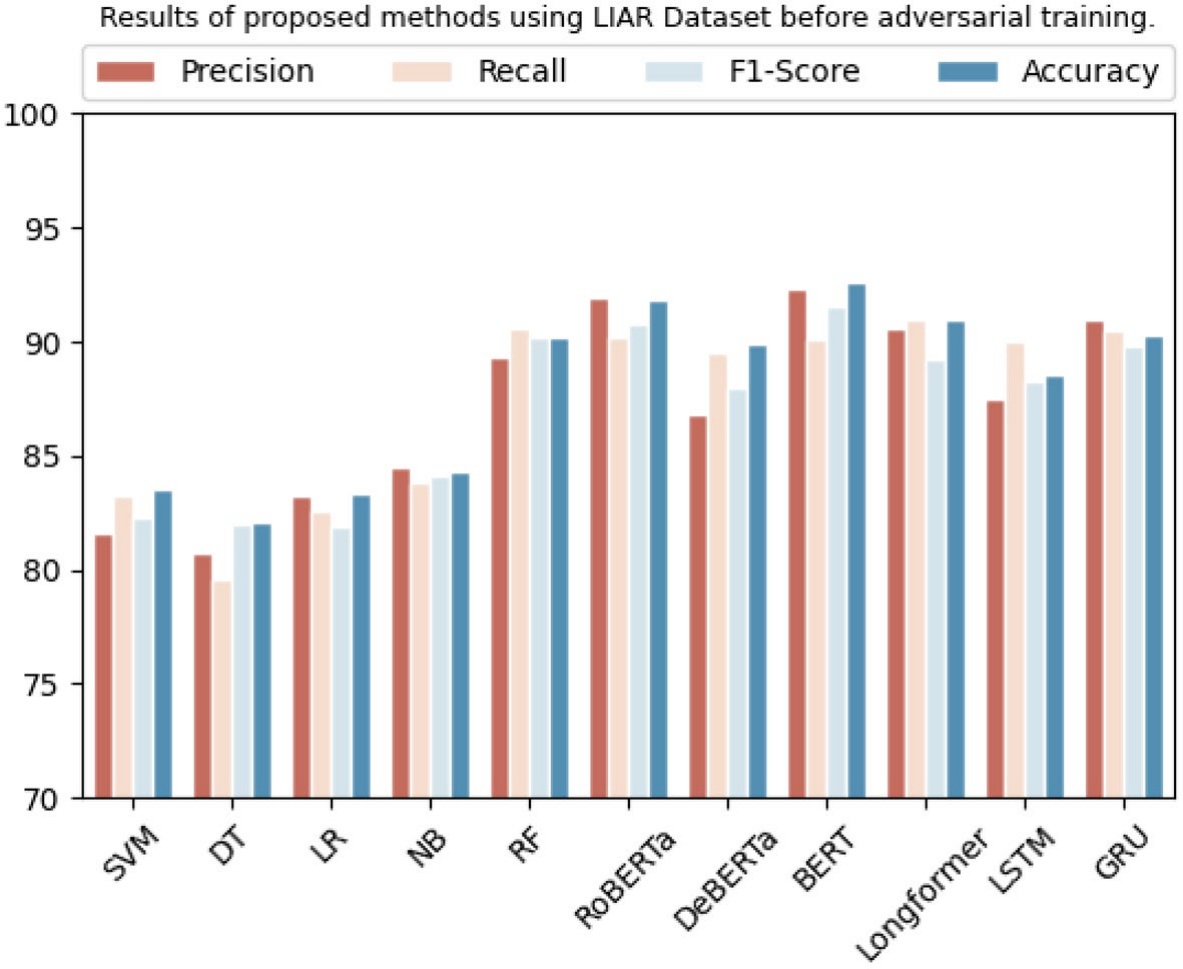
Graphic representation of results on baseline methods using LIAR Dataset.

On the LIAR dataset, SVM, decision tree, logistic regression, Naive Bayes, and random forest achieved accuracy of 83.5%, 82.1%, 83.3%, 84.3%, and 90.2%. In contrast, RoBERTa, BERT, Longformer, DeBERTa, LSTM, and GRU achieved accuracies of 91.8%, 92.6%, 95.6%, 94.9%, 89.6%, and 90.3%, respectively. Table 8 shows the models' overall results, and their graphical representation is shown in Fig. 5.

**Table 8:** Results of proposed methods using LIAR dataset before adversarial training.

Dataset	Methodology	Precision	Recall	F1-score	Accuracy
LIAR	SVM	81.5%	83.2%	82.3%	83.5%
	Decision tree	80.7%	79.5%	81.9%	82.1%
	Logistic regression	83.2%	82.5%	81.9%	83.3%
	Naive Bayes	84.5%	83.8%	84.1%	84.3%
	Random forest	89.3%	90.5%	90.1%	90.2%
	RoBERTa	91.9%	90.2%	90.7%	91.8%
	DeBERTa	86.8%	89.5%	87.9%	89.9%
	BERT	92.3%	90.1%	91.5%	92.6%
	Longformer	90.5%	91.0%	89.2%	90.6%
	LSTM	87.5%	90.0%	88.2%	88.6%
	GRU	91.0%	90.5%	89.8%	90.3%

**Figure 6:**
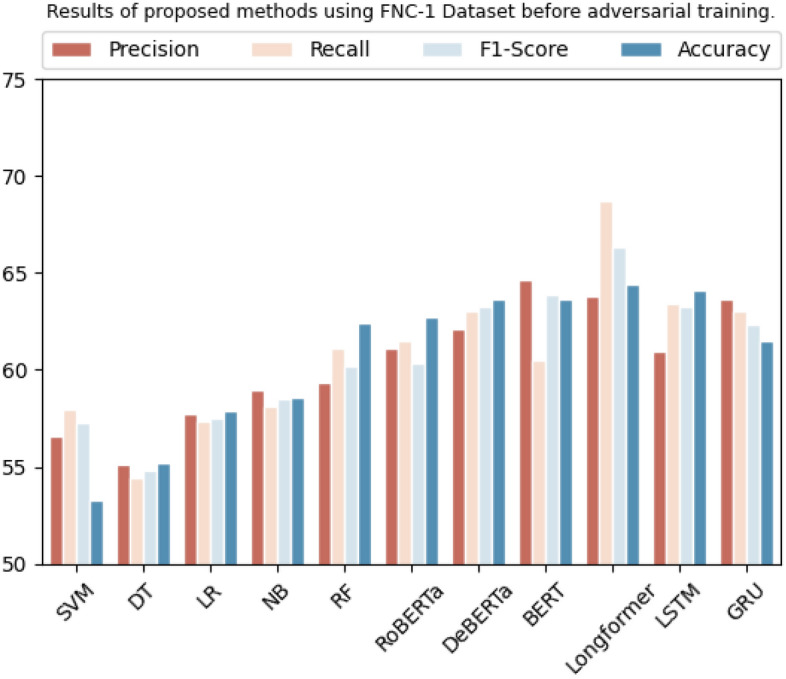
Graphic representation of results on baseline methods using FNC-1 dataset.

On the FNC-1 dataset, SVM, decision tree, logistic regression, Naive Bayes, and random forest achieved an accuracy of 53.2%, 55.2%, 57.8%, 58.6%, and 58.6%, respectively. Transformer-based algorithms like RoBERTa, BERT, DeBERTa, and Longformer achieved accuracy of 62.4%, 63.6%, 62.7%, and 64.4%, respectively. LSTM and GRU achieved an accuracy of 64.0% and 61.5% on the same dataset, respectively. Table 9 and Fig. 6 show their overall results in tabular and graphical form.

**Table 9:** Results of proposed methods using the fNC-1 dataset before adversarial training.

Dataset	Methodology	Precision	Recall	F1-score	Accuracy
FNC-1	SVM	57.5%	57.9%	57.2%	53.2%
	Decision tree	54.1%	54.3%	54.7%	55.2%
	Logistic regression	57.7%	57.3%	57.51%	57.8%
	Naive Bayes	59.9%	58.1%	58.52%	58.6%
	Random forest	61.3%	61.0%	60.1%	58.6%
	RoBERTa	61.1%	61.5%	60.3%	62.4%
	DeBERTa	63.1%	63.0%	63.2%	62.7%
	BERT	64.6%	60.4%	63.8%	63.6%
	Longformer	63.7%	68.6%	66.3%	64.4%
	LSTM	60.9%	63.4%	63.2%	64.0%
	GRU	63.6%	63.0%	62.3%	61.5%

#### Results after adversarial training

Once the baseline models have produced results, adversarial samples are generated on word embedding levels using the fast gradient sign method (FGSM). These samples are then utilized to train the models. Using perturbed samples during training significantly improved model robustness and led to enhanced performance. By incorporating adversarial samples into the training process, models became better equipped to handle unexpected scenarios and produced more accurate results in real-world applications.

The "Random Political News” dataset is evaluated using several ML algorithms. SVM demonstrated a precision of 82.3%, recall of 83.4%, F1 score of 83.1%, and accuracy of 84.4%. Similarly, the Decision Tree yielded a precision of 81.2%, recall of 80.1%, F1 score of 83.6%, and accuracy of 83.7%. Logistic Regression showed a precision of 84.1%, recall of 83.1%, F1 score of 83.4%, and accuracy of 84.88%. Naive Bayes yielded a precision of 85.2%, recall of 84.9%, F1 score of 85.1%, and accuracy of 85%. Random Forest achieved a precision of 90%, recall of 92.3%, F1 score of 91%, and accuracy of 92.7%.

**Figure 7:**
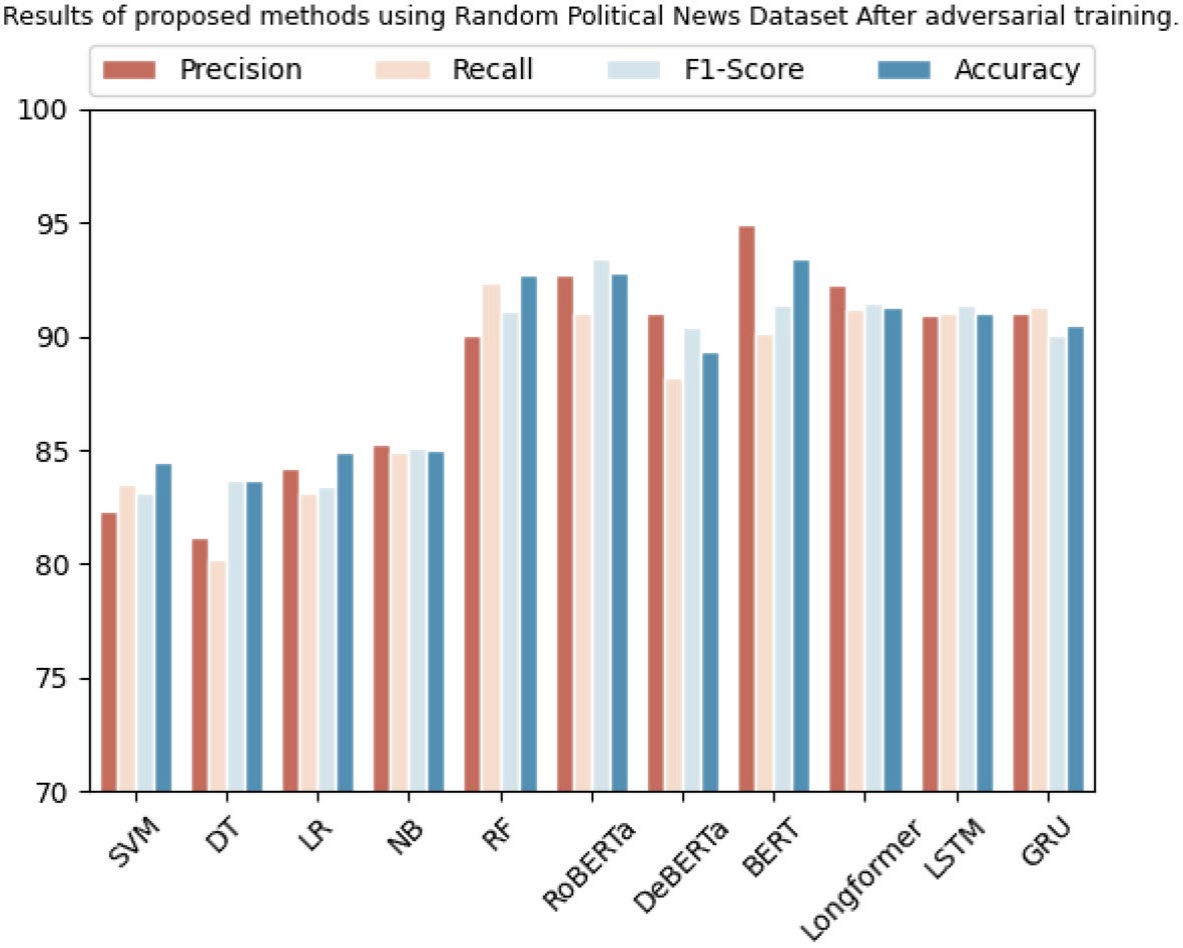
Graphical representation of results after adversarial training using random political news dataset.

On the other hand, Transformer-based models such as RoBERTa demonstrated a precision of 92.7%, recall of 91%, F1 score of 93.4%, and accuracy of 92.8%. DeBERTa yielded a precision of 91.0%, recall of 88.2%, F1 score of 90.4%, and accuracy of 89.33%. BERT showed a precision of 94.9%, recall of 90.1%, F1 score of 91.3%, and accuracy of 93.43%. Longformer yielded a precision of 92.2%, recall of 91.1%, F1 score of 91.4%, and accuracy of 91.3%. LSTM and GRU achieved a precision of 90.9% and 91%, recall of 91% and 91.3%, F1 score of 91.3% and 90%, and accuracy of 91% and 90.5%, respectively as shown in Table 10. While Fig. 7 shows the comparison of results in graphical form.

**Table 10:** Results of proposed methods using random political news dataset after adversarial training.

Dataset	Methodology	Precision	Recall	F1-score	Accuracy
Random political news	SVM	82.3%	83.4%	83.1%	84.4%
	Decision tree	81.2%	80.1%	83.6%	83.7%
	Logistic regression	84.1%	83.1%	83.4%	84.88%
	Naive Bayes	85.2%	84.9%	85.1%	85%
	Random forest	90%	92.3%	91%	92.7%
	RoBERTa	92.7%	91%	93.4%	92.8%
	DeBERTa	91.0%	88.2%	90.4%	89.33%
	BERT	94.9%	90.1%	91.3%	93.43%
	Longformer	92.2%	91.1%	91.4%	91.3%
	LSTM	90.9%	91%	91.3%	91%
	GRU	91%	91.3%	90%	90.5%

**Figure 8:**
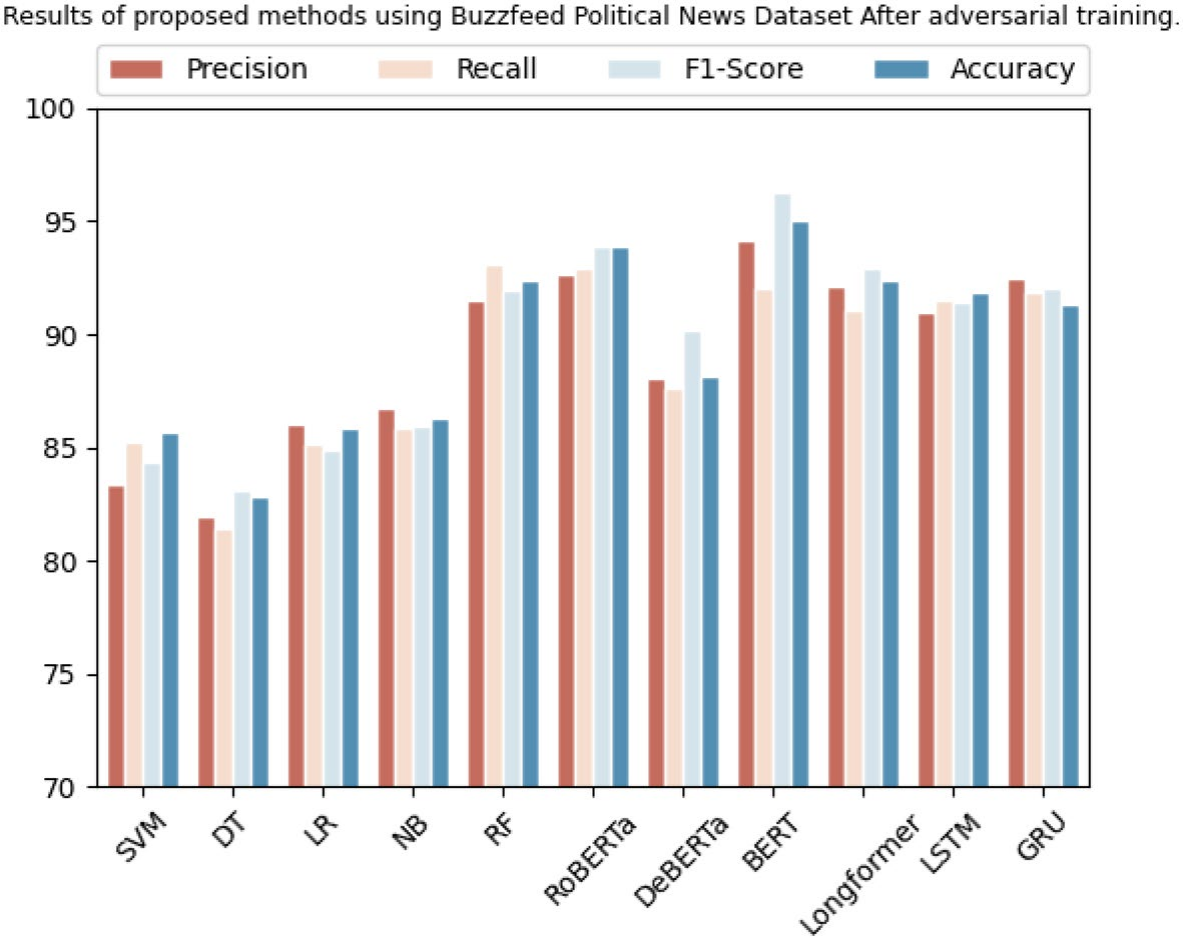
Graphical representation of results after adversarial training using Buzzfeed political news dataset.

The BuzzFeed political news dataset is used to evaluate several ML algorithms. SVM, decision tree, logistic regression, Naive Bayes, and random forest algorithms are all tested. The metrics used to evaluate these algorithms are precision, recall, F1-score, and accuracy as shown in Table 11. These algorithms achieved precision, recall, F1-score, and accuracy scores of 83.3%, 85.1%, 84.3%, and 85.6%, 82.0%, 81.3%, 83.0%, and 82.8%, 86.0%, 85.1%, 84.9%, and 85.8%, 86.7%, 85.8%, 85.9%, and 86.3%, and 91.5%, 93.0%, 91.9%, and 92.4%, respectively. Figure 8 shows the graphical results on BuzzFeed political news dataset.

**Table 11:** Results of proposed methods using Buzzfeed political news dataset after adversarial training.

Dataset	Methodology	Precision	Recall	F1-score	Accuracy
BuzzFeed political news	SVM	83.3%	85.1%	84.3%	85.6%
	Decision tree	82.0%	81.3%	83.0%	82.8%
	Logistic regression	86.0%	85.1%	84.9%	85.8%
	Naive Bayes	86.7%	85.8%	85.9%	86.3%
	Random forest	91.5%	93.0%	91.9%	92.4%
	RoBERTa	92.6%	92.9%	93.8%	93.9%
	DeBERTa	88.0%	87.6%	90.1%	88.1%
	BERT	94.1%	92.0%	96.2%	95.0%
	Longformer	92.1%	91.0%	92.9%	92.4%
	LSTM	91.0%	91.5%	91.4%	91.8%
	GRU	92.5%	91.8%	92.0%	91.3%

Transformer-based algorithms such as RoBERTa, DeBERTa, BERT, and Longformer are also evaluated. RoBERTa achieved precision, recall, F1-score, and accuracy scores of 92.6%, 92.9%, 91.1%, and 93.9%, DeBERTa achieved 88.0%, 87.6%, 90.1%, and 88.1%, BERT achieved 94.1%, 92.0%, 96.2%, and 95.0%, and Longformer achieved 92.1%, 91.0%, 92.9%, and 92.4%, respectively. LSTM and GRU are also evaluated and achieved precision scores of 91.0% and 92.5%, recall scores of 91.5% and 91.8%, F1-scores of 91.4% and 92.0%, and accuracy scores of 91.8% and 91.3%, respectively.

**Figure 9:**
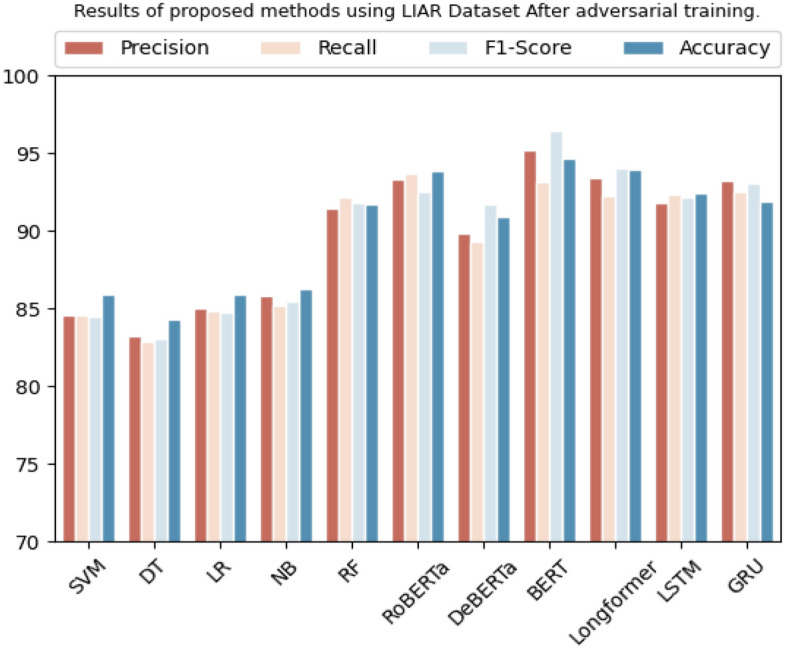
Graphical representation of results after adversarial training using LIAR dataset.

On LIAR dataset, SVM, decision tree, logistic regression, Naive Bayes, and random forest achieved accuracies of 85.9%, 84.4%, 85.8%, 86.3%, and 91.7%, respectively. On the other hand, Transformer-based models like RoBERTa, BERT, Longformer, DeBERTa, LSTM, and GRU achieved significantly higher accuracies of 93.8%, 94.6%, 93.9%, 90.9%, 92.4%, and 91.9%, respectively. The models with the highest accuracy are BERT and RoBERTa, with accuracies of 94.6% and 93.8%, respectively. The models with the highest precision are BERT and Longformer, with precision scores of 95.2% and 93.4%, respectively. The models with the highest recall scores are RoBERTa and BERT, with recall scores of 93.7% and 93.1%, respectively. The models with the highest F1-scores are BERT and LSTM, with F1-scores of 96.4% and 92.1%, respectively. Overall, the results show that Transformer-based models outperformed traditional ML models on the LIAR dataset as it can be clearly seen in Table 12 and Fig. 9.

**Table 12:** Results of proposed methods using Liar dataset after adversarial training.

Dataset	Methodology	Precision	Recall	F1-score	Accuracy
LIAR	SVM	84.5%	84.5%	84.5%	85.9%
	Decision tree	83.2%	82.8%	83.0%	84.4%
	Logistic regression	85.0%	84.8%	84.7%	85.8%
	Naive Bayes	85.8%	85.2%	85.4%	86.3%
	Random forest	91.4%	92.1%	91.7%	91.7%
	RoBERTa	93.3%	93.7%	92.5%	93.8%
	DeBERTa	89.8%	89.3%	91.7%	90.9%
	BERT	95.2%	93.1%	96.4%	94.6%
	Longformer	93.4%	92.2%	94.0%	93.9%
	LSTM	91.8%	92.3%	92.1%	92.4%
	GRU	93.2%	92.5%	93.0%	91.9%

**Figure 10:**
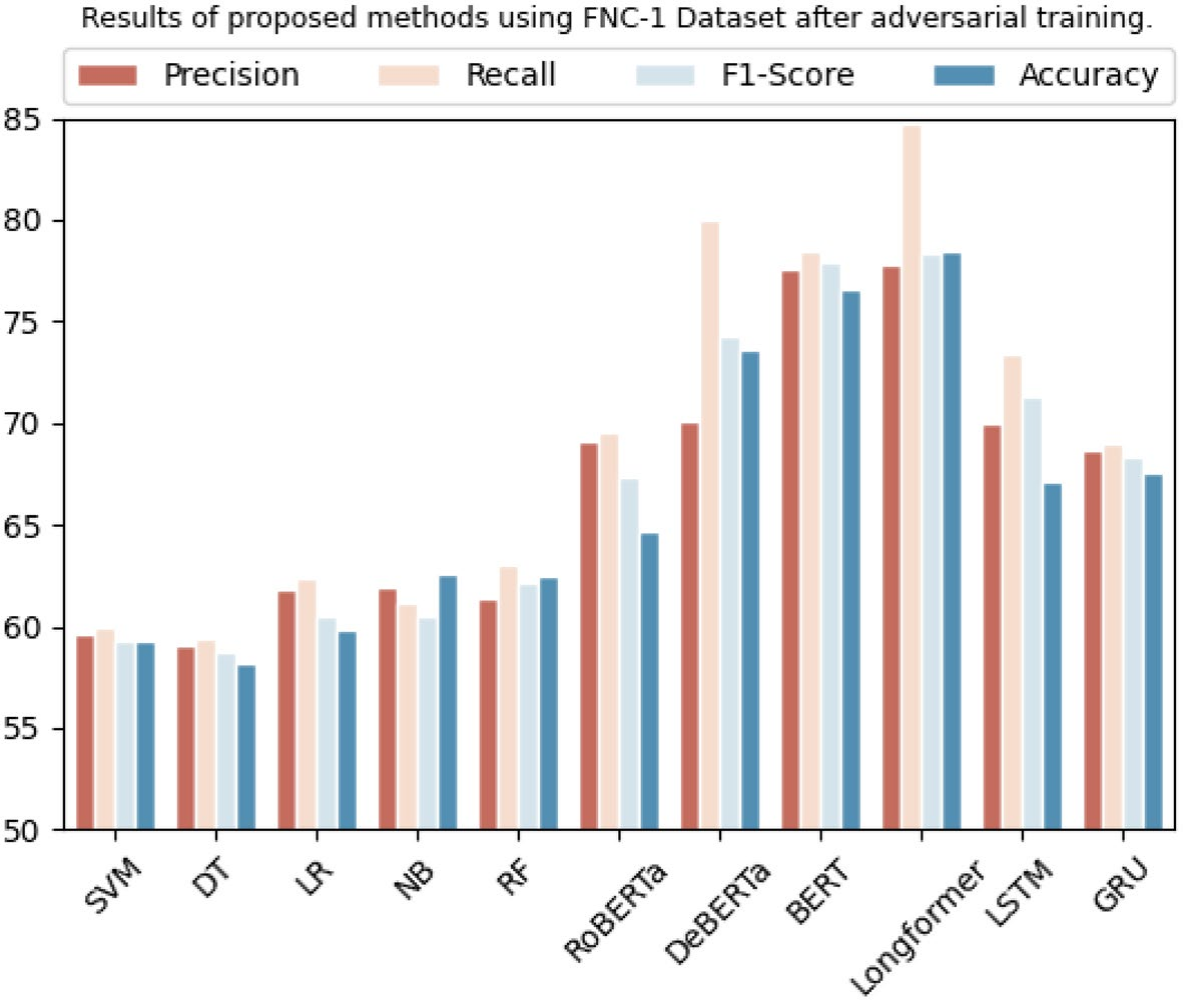
Graphical representation of results after adversarial training using FNC-1 dataset.

On the FNC-1 dataset, several classification models are evaluated. The SVM, decision tree, logistic regression, Naive Bayes, and random forest achieved an accuracy of 59.2%, 58.2%, 59.8%, 62.6%, and 62.4%, respectively. Among the transformer-based algorithms, RoBERTa, BERT, DeBERTa, and Longformer achieved an accuracy of 64.7%, 73.7%, 76.6%, and 78.4%, respectively. The LSTM and GRU models attained an accuracy of 67.0% and 67.5%, respectively. Overall, the results are improved by 3% to 14%, as can be seen in Table 13. Additionally, precision, recall, and F1-score are calculated for each model based on accuracy. Overall, the results showed that transformer-based algorithms achieved the highest accuracy, while Naive Bayes and Random Forest achieved high accuracy among traditional models. Figure 10 depicts the comparison of these methods.

**Table 13:** Results of proposed methods using the FNC-1 dataset after adversarial training.

Dataset	Methodology	Precision	Recall	F1-score	Accuracy
FNC-1	SVM	59.5%	59.9%	59.2%	59.2%
	Decision tree	59.1%	59.3%	58.7%	58.2%
	Logistic regression	61.7%	62.3%	60.5%	59.8%
	Naive Bayes	61.9%	61.1%	60.5%	62.6%
	Random forest	61.3%	63.0%	62.1%	62.4%
	RoBERTa	69.1%	69.5%	67.3%	64.7%
	BERT	70.1%	80.0%	74.2%	73.7%
	DeBERTa	77.6%	78.4%	77.8%	76.6%
	Longformer	77.7%	84.6%	78.3%	78.4%
	LSTM	69.9%	73.4%	71.2%	67.0%
	GRU	68.6%	69.0%	68.3%	67.5%

#### Comparative analysis

Table 14 compares the precision, recall, F1-score and accuracy of different methods used in various studies to detect fake news on other datasets. This table also provides the aim and methods used in previous studies. Our study used BERT with adversarial training and achieved the highest accuracy on three out of four datasets—Random Political News (93.43%), Buzzfeed Political News (95%) and LIAR (94.6%). Our model outperformed other studies that used methods such as a Linguistic feature-based framework, K mean clustering, K-nearest Neighbor, an attention-enabled neural architecture, a Deep ensemble model using sequential DL, and combined CNN and DNN with SCM.

**Table 14:** Comparative analysis of our proposed framework results with recent studies.

Authors, year of study	Methods	Dataset				Aim	Accuracy	Precision	Recall	F1-score
		Random political news	Buzzfeed political news	LIAR	FNC-1					
Baseline methods										
Machine learning										
Yazdi, K.M. et al.^24^, 2020	K mean clustering		✓	✓		To improve fake news detection using ML methods	–	95.34%	–	–
Kareem, I. et al. 2019^25^	K-nearest neighbor					To classify fake news articles on Pakistani social media	70%	70%	65%	62%
Choudhury, D. 2023^26^	Support vector machine			✓		To detect fake news on social network platforms using traditional ML methods	61%	62%	79%	69%
Ours	Random forest	✓	✓	✓	✓	To develop a framework for robust and generalized fake news classification incorporated with emotes and slang words	91.1%	90.0%	91.7%	90.6%
Deep learning										
Garg, S. et al.^27^, 2022	Linguistic feature-based framework	✓	✓			To automate the system for fake news classification using a linguistic feature-based method	93%	96%	92%	94%
Jain, V. et al.^28^, 2021	An attention-enabled neural architecture			✓		To detect fake news by developing a neural network architecture equipped with an attention mechanism	46.3%	41.6%	57.6%	48.3%
Ali, A.M. et al. ^29^, 2022	Deep ensemble model using sequential DL			✓		To enhance the accuracy of fake news detection through the introduction of a deep ensemble model employing sequential deep learning methodologies	75%	84%	87%	85%
Tariq, A. et al.^20^, 2022	Adversarial training using FGSM	✓	✓				–	81.3%	80%	79.3%
Zainab, A. et al.^30^, 2022	Combine CNN and DNN with SCM				✓		84.6%	84.6%	84.6%	84.6%
Ours	BERT	✓	✓	✓	✓	To develop a framework for robust and generalized fake news classification incorporated with emotes and slang words	93.6%	92.2%	90.3%	94.5%
After adversarial training										
Deep learning										
Tariq, A. et al.^20^, 2022	Adversarial training using FGSM	✓	✓			To classify short and long text based fake news	–	84.6%	80.%	80.5%
Liu, X. et al.^31^, 2023	Hierarchical neural network and gradient reversal framework					To propose an adversarial training method for text classification	–	67.5%	–	–
Ours	BERT	✓	✓	✓	✓	To develop a framework for robust and generalized fake news classification incorporated with emotes and slang words	95%	94.1%	92.0%	96.2%

## Limitations

The reliance on a trial-and-error method for selecting the alpha value in the fast gradient sign method (FGSM) for adversarial training introduces subjectivity and may not guarantee optimal performance. Moreover, the study's focus solely on textual data rather than incorporating multimodal data limits the generalizability of the findings and may overlook potential information in real-world scenarios where multiple modalities are present. To enhance the robustness and applicability of the research, future studies could explore automated methods for alpha value selection and incorporate multimodal data for a more comprehensive analysis of adversarial training methodologies.

## Conclusion

The classification of fake news is an essential task in today's society, given the prevalence of misleading information in various forms of media. This study focused on achieving this task by proposing a framework named as “ANN: Adversarial News Net”. Adversarial training is introduced in this proposed framework as a generalization to enhance the robustness of our model. The model is trained on perturbed samples to improve performance on unseen datasets. Additionally, emoticons were extracted from the dataset and incorporated into the model to enhance results. The models were applied to four publicly available datasets: Random Political News, Buzzfeed Political News, LIAR, and FNC-1. Results showed that adversarial training significantly improved the performance of all models compared to baseline methods, making them more robust. Overall, this study offers valuable insights into the effectiveness of using adversarial training in fake news classification, emphasizing the importance of ongoing research to combat misinformation.

## Data Availability

The datasets used during the current study are available from the corresponding author upon reasonable request.
